# Enhancing clinical skills in pediatric trainees: a comparative study of ChatGPT-assisted and traditional teaching methods

**DOI:** 10.1186/s12909-024-05565-1

**Published:** 2024-05-22

**Authors:** Hongjun Ba, Lili zhang, Zizheng Yi

**Affiliations:** 1 Department of Pediatric Cardiology, Heart Center, First Affiliated Hospital of Sun Yat-sen University, 58# Zhongshan Road 2, Guangzhou, 510080 China; 2 Key Laboratory on Assisted Circulation, Ministry of Health, 58# Zhongshan Road 2, Guangzhou, 510080 China

**Keywords:** ChatGPT, Pediatric training, Clinical skills, Medical education, AI in healthcare, Mini-clinical evaluation exercise, Interactive learning

## Abstract

**Background:**

As artificial intelligence (AI) increasingly integrates into medical education, its specific impact on the development of clinical skills among pediatric trainees needs detailed investigation. Pediatric training presents unique challenges which AI tools like ChatGPT may be well-suited to address.

**Objective:**

This study evaluates the effectiveness of ChatGPT-assisted instruction versus traditional teaching methods on pediatric trainees’ clinical skills performance.

**Methods:**

A cohort of pediatric trainees (*n* = 77) was randomly assigned to two groups; one underwent ChatGPT-assisted training, while the other received conventional instruction over a period of two weeks. Performance was assessed using theoretical knowledge exams and Mini-Clinical Evaluation Exercises (Mini-CEX), with particular attention to professional conduct, clinical judgment, patient communication, and overall clinical skills. Trainees’ acceptance and satisfaction with the AI-assisted method were evaluated through a structured survey.

**Results:**

Both groups performed similarly in theoretical exams, indicating no significant difference (*p* > 0.05). However, the ChatGPT-assisted group showed a statistically significant improvement in Mini-CEX scores (*p* < 0.05), particularly in patient communication and clinical judgment. The AI-teaching approach received positive feedback from the majority of trainees, highlighting the perceived benefits in interactive learning and skill acquisition.

**Conclusion:**

ChatGPT-assisted instruction did not affect theoretical knowledge acquisition but did enhance practical clinical skills among pediatric trainees. The positive reception of the AI-based method suggests that it has the potential to complement and augment traditional training approaches in pediatric education. These promising results warrant further exploration into the broader applications of AI in medical education scenarios.

## Introduction

The introduction of ChatGPT by OpenAI in November 2022 marked a watershed moment in educational technology, heralded as the third major innovation following Web 2.0’s emergence over a decade earlier [1] and the rapid expansion of e-learning driven by the COVID-19 pandemic [2]. In medical education, the integration of state-of-the-art Artificial Intelligence (AI) has been particularly transformative for pediatric clinical skills training—a field where AI is now at the forefront.

Pediatric training, with its intricate blend of extensive medical knowledge and soft skills like empathetic patient interaction, is pivotal for effective child healthcare. The need for swift decision-making, especially in emergency care settings, underscores the specialty’s complexity. Traditional teaching methods often fall short, hindered by logistical challenges and difficulties in providing a standardized training experience. AI tools such as ChatGPT offer a promising solution, with their ability to simulate complex patient interactions and thus improve pediatric trainees’ communication, clinical reasoning, and decision-making skills across diverse scenarios [3, 4].

ChatGPT’s consistent, repeatable, and scalable learning experiences represent a significant advancement over traditional constraints, such as resource limitations and standardization challenges [5], offering a new paradigm for medical training. Its proficiency in providing immediate, personalized feedback could revolutionize the educational journey of pediatric interns. Our study seeks to investigate the full extent of this potential revolution, employing a mixed-methods approach to quantitatively and qualitatively measure the impact of ChatGPT on pediatric trainees’ clinical competencies.

Despite AI’s recognized potential within the academic community, empirical evidence detailing its influence on clinical skills development is limited [6]. Addressing this gap, our research aims to contribute substantive insights into the efficacy of ChatGPT in enhancing the clinical capabilities of pediatric trainees, establishing a new benchmark for the intersection of AI and medical education.

## Participants and methods

### Participants

Our study evaluated the impact of ChatGPT-assisted instruction on the clinical skills of 77 medical interns enrolled in Sun Yat-sen University’s five-year program in 2023. The cohort, consisting of 42 males and 35 females, was randomly allocated into four groups based on practicum rotation, using a computer-generated randomization list. Each group, composed of 3–4 students, was assigned to either the ChatGPT-assisted or traditional teaching group for a two-week pediatric internship rotation. Randomization was stratified by baseline clinical examination scores to ensure group comparability.

## Methods

### Study design

**Fig. 1:**
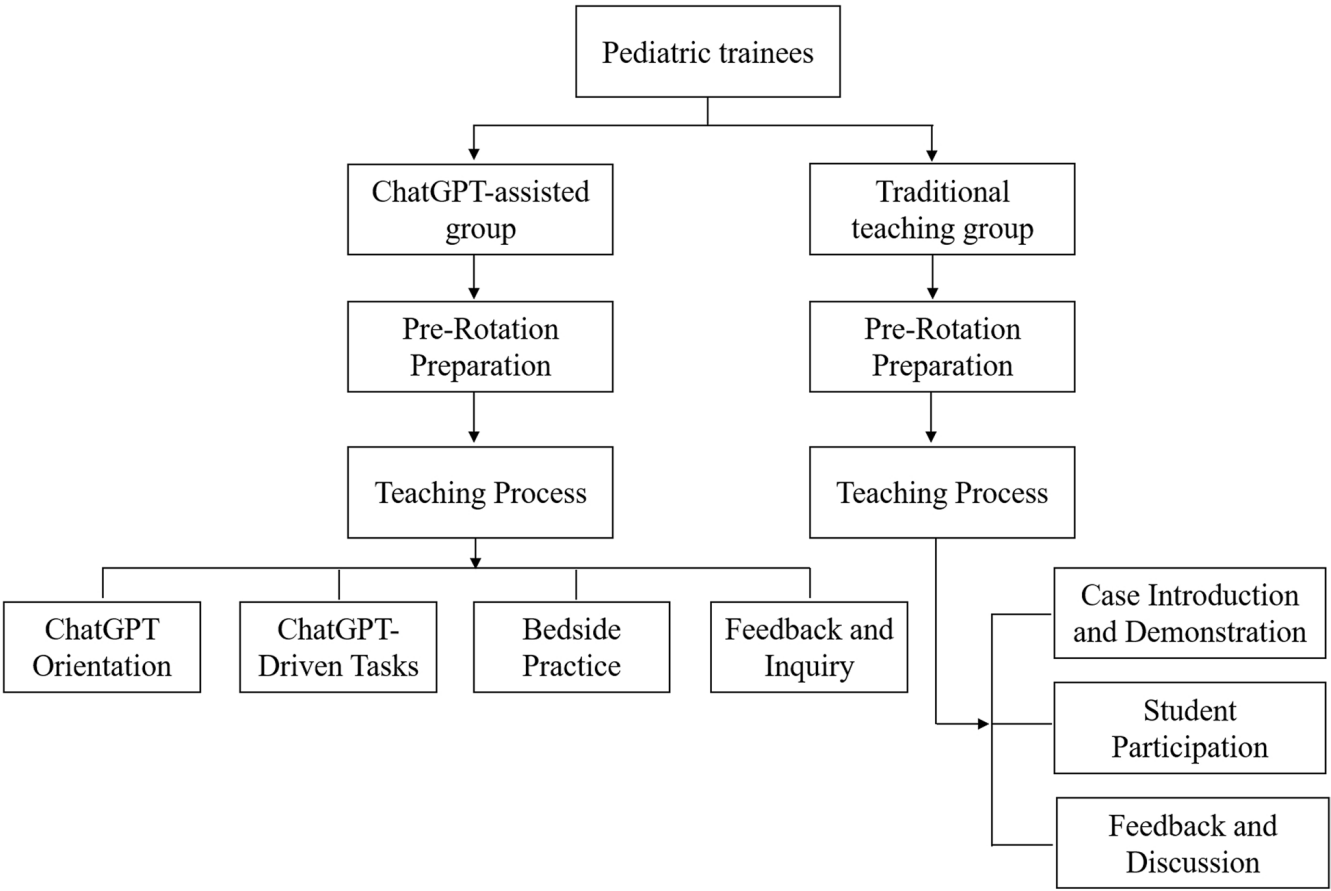
Study design and flow chart

A controlled experimental design was implemented with blind assessment. The interns were randomly assigned to the ChatGPT-assisted group (39 students) or the traditional group (38 students), with no significant differences in gender, age, or baseline clinical examination scores (*p* > 0.05). The ChatGPT-assisted group received instruction supplemented with ChatGPT version 4.0, while the traditional group received standard bedside teaching (as depicted in Fig. 1). Both groups encountered identical clinical case scenarios involving common pediatric conditions: Kawasaki disease, gastroenteritis, congenital heart disease, nephrotic syndrome, bronchopneumonia, and febrile convulsion. All interns had equal access to the same teaching materials, instructors, and intensity of courses. The core textbook was the 9th edition of “Pediatrics” published by the People’s Medical Publishing House. Ethical approval was obtained from the institutional review board, and informed consent was secured, with special attention to privacy concerns due to the involvement of pediatric patient data.

## Instructional implementation

### Traditional teaching group

#### Pre-rotation preparation

Instructors designed typical cases representing common pediatric diseases and updated knowledge on the latest diagnostic and therapeutic advancements. They developed multimedia presentations detailing the presentation, diagnostic criteria, and treatment plans for each condition.

#### Teaching process

The teaching method during the rotation was divided into three stages:

##### Case introduction and demonstration

Instructors began with a detailed introduction of clinical cases, explaining diagnostic reasoning and emphasizing key aspects of medical history-taking and physical examination techniques.

##### Student participation

Students then conducted patient interviews and physical assessments independently, with the instructor observing. For pediatric patients, particularly infants, history was provided by the guardians.

##### Feedback and discussion

At the end of each session, instructors provided personalized feedback on student performance and answered questions, fostering an interactive learning environment.

### ChatGPT-assisted teaching group

#### Pre-rotation preparation

Educators prepared structured teaching plans focusing on common pediatric diseases and representative cases. The preparation phase involved configuring ChatGPT (version 4.0) settings to align with the educational objectives of the rotation.

#### Teaching process

The rotation was executed in four consecutive steps:

##### ChatGPT orientation

Students were familiarized with the functionalities and potential educational applications of ChatGPT version 4.0.

##### ChatGPT-driven tasks

In our study, ChatGPT version 4.0 was used as a supplementary educational tool within the curriculum. Students engaged with the AI to interactively explore dynamically generated clinical case vignettes based on pediatric medicine. These vignettes encompassed clinical presentations, history taking, physical examinations, diagnostic strategies, differential diagnoses, and treatment protocols, allowing students to query the AI to enhance their understanding of various clinical scenarios.

Students accessed clinical vignettes in both text and video formats, with video particularly effective in demonstrating physical examination techniques and communication strategies with guardians, thereby facilitating a more interactive learning experience.

ChatGPT initially guided students in forming assessments, while educators critically reviewed their work, providing immediate, personalized feedback to ensure proper development of clinical reasoning and decision-making skills. This blend of AI and direct educator involvement aimed to improve learning outcomes by leveraging AI’s scalability alongside expert educators’ insights.

##### Bedside clinical practice

Students practiced history-taking and physical examinations at the patient’s bedside, with information about infants provided by their guardians.

##### Feedback and inquiry

Instructors offered feedback on performance and addressed student queries to reinforce learning outcomes.

#### Assessment methods

The methods used to evaluate the interns’ post-rotation performance included three assessment tools:

##### Theoretical knowledge exam

Both groups completed the same closed-book exam to test their pediatric theoretical knowledge, ensuring consistency in cognitive understanding assessment.

##### Mini-CEX assessment

The Mini-CEX has been widely recognized as an effective and reliable method for assessing clinical skills [7, 8]. Practical skills were evaluated using the Mini-CEX, which involved students taking histories from parents of pediatric patients and conducting physical examinations on infants, supervised by an instructor. Mini-CEX scoring utilized a nine-point scale with seven criteria, assessing history-taking, physical examination, professionalism, clinical judgment, doctor-patient communication, organizational skills, and overall competence.

##### History taking

This assessment measures students’ ability to accurately collect patient histories, utilize effective questioning techniques, respond to non-verbal cues, and exhibit respect, empathy, and trust, while addressing patient comfort, dignity, and confidentiality.

##### Physical examination

This evaluates students on informing patients about examination procedures, conducting examinations in an orderly sequence, adjusting examinations based on patient condition, attending to patient discomfort, and ensuring privacy.

##### Professionalism

This assesses students’ demonstration of respect, compassion, and empathy, establishment of trust, attention to patient comfort, maintenance of confidentiality, adherence to ethical standards, understanding of legal aspects, and recognition of their professional limits.

##### Clinical judgment

This includes evaluating students’ selection and execution of appropriate diagnostic tests and their consideration of the risks and benefits of various treatment options.

##### Doctor-patient communication

This involves explaining test and treatment rationales, obtaining patient consent, educating on disease management, and discussing issues effectively and timely based on disease severity.

##### Organizational efficiency

This measures how students prioritize based on urgency, handle patient issues efficiently, demonstrate integrative skills, understand the healthcare system, and effectively use resources for optimal service.

##### Overall competence

This assesses students on judgment, integration, and effectiveness in patient care, evaluating their overall capabilities in caring and efficiency.

The scale ranged from below expectations (1–3 points), meeting expectations (4–6 points), to exceeding expectations (7–9 points). To maintain assessment consistency, all Mini-CEX evaluations were conducted by a single assessor.

##### ChatGPT method feedback survey

Only for the ChatGPT-assisted group, the educational impact of the ChatGPT teaching method was evaluated post-rotation through a questionnaire. This survey used a self-assessment scale with a Cronbach’s Alpha coefficient of 0.812, confirming its internal consistency and reliability. Assessment items involved active learning engagement, communication skills, empathy, retention of clinical knowledge, and improvement in diagnostic reasoning. Participant satisfaction was categorized as (1) very satisfied, (2) satisfied, (3) neutral, or (4) dissatisfied.

### Statistical analysis

Data were analyzed using R software (version 4.2.2) and SPSS (version 26.0). Descriptive statistics were presented as mean ± standard deviation (x ± s), and independent t-tests were performed to compare groups. Categorical data were presented as frequency and percentage (n[%]), with chi-square tests applied where appropriate. A *P*-value of < 0.05 was considered statistically significant. All assessors of the Mini-CEX were blinded to the group assignments to minimize bias.

## Results

### Theoretical knowledge exam scores for both groups of trainees

The theoretical knowledge exam revealed comparable results between the two groups, with the ChatGPT-assisted group achieving a mean score of 92.21 ± 2.37, and the traditional teaching group scoring slightly higher at 92.38 ± 2.68. Statistical analysis using an independent t-test showed no significant difference in the exam scores (t = 0.295, *p* = 0.768), suggesting that both teaching methods similarly supported the trainees’ theoretical learning.

### Mini-CEX evaluation results for both groups of trainees

**Fig. 2:**
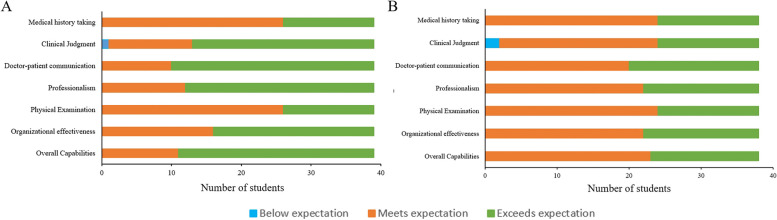
The scale scores of Mini-CEX assessment between the two groups. **A**: ChatGPT-assisted group; **B**: Traditional teaching group

All trainees completed the Mini-CEX evaluation in 38 ± 0.5 min on average, with immediate post-evaluation feedback averaging 5.8 ± 0.6 min per student. The ChatGPT group demonstrated statistically significant improvement in professional conduct, clinical judgment, patient communication, and overall clinical skills compared to the traditional group. A detailed comparison of the CEX scoring for both student groups is presented in Table 1; Fig. 2.

**Table 1:** The scale scores of Mini-CEX assessment between the two groups

Items	ChatGPT-assisted group, cases (%)	Traditional teaching group, cases (%)	*P*-value
Total cases	39(100%)	38(100%)	
Medical history taking			0.814
Meets expectation	26(66.7%)	24(63.2%)	
Exceeds expectation	13(33.3%)	14(36.8%)	
Clinical Judgment			0.032
Below expectation	1(2.6%)	2(5.3%)	
Meets expectation	12(30.8%)	22(57.9%)	
Exceeds expectation	26(66.6%)	14(36.8%)	
Doctor-patient communication			0.02
Meets expectation	10(25.6%)	20(52.6%)	
Exceeds expectation	29(74.4%)	18(47.4%)	
Professionalism			0.022
Meets expectation	12(30.8%)	22(57.9%)	
Exceeds expectation	27(69.2%)	16(42.1%)	
Physical Examination			0.814
Meets expectation	26(66.7%)	24(63.2%)	
Exceeds expectation	13(33.3%)	14(36.8%)	
Organizational effectiveness			0.174
Meets expectation	16(41.0%)	22(57.9%)	
Exceeds expectation	23(59.0%)	16(42.1%)	
Overall Capabilities			0.006
Meets expectation	11(28.2%)	23(60.5%)	
Exceeds expectation	28(71.8%)	15(39.5%)	

### Satisfaction survey results of trainees in the ChatGPT-assisted teaching

Feedback from the trainees regarding the ChatGPT-assisted teaching method was overwhelmingly positive. High levels of satisfaction and interest were reported, with no instances of dissatisfaction noted. The summary of these findings, including specific aspects of the teaching method that were rated highly by the students, is detailed in Table 2.

**Table 2:** Students satisfaction evaluation of the ChatGPT-assisted group

Items	Very satisfied(*n*)	Satisfied(*n*)	Neutral(*n*)	Dissatisfied(*n*)	Satisfaction (%)
Improving learning efficiency	38	1	0	0	100
Stimulating an interest	37	1	1	0	97.4
Improving communication skills	35	2	2	0	94.9
Deeper understanding of clinical skills and theoretical knowledge	36	1	2	0	94.9
Enhancing students’ self-learning abilities	38	1	0	0	100
Helping to achieve learning objectives	36	2	1	0	97.4

## Discussion

The integration of ChatGPT into pediatric medical education represents a significant stride in leveraging artificial intelligence (AI) to enhance the learning process. Our findings suggest that while AI does not substantially alter outcomes in theoretical knowledge assessments, it plays a pivotal role in the advancement of clinical competencies.

The parity in theoretical examination scores between the ChatGPT-assisted and traditionally taught groups indicates that foundational medical knowledge can still be effectively acquired through existing educational frameworks. This underscores the potential of ChatGPT as a complementary, rather than a substitutive, educational instrument [9, 10].

Mini-CEX evaluations paint a different picture, revealing the ChatGPT group’s superior performance in clinical realms. These competencies are crucial for the comprehensive development of a pediatrician and highlight the value of an interactive learning environment in bridging the gap between theory and practice [11, 12].

The unanimous satisfaction with ChatGPT-assisted learning points to AI’s capacity to enhance student engagement. This positive response could be attributed to the personalized and interactive nature of the AI experience, catering to diverse learning styles [13, 14]. However, it is critical to consider the potential for overreliance on technology and the need for maintaining an appropriate balance between AI and human interaction in medical training.

The ChatGPT group’s ascendency in clinical skillfulness could be a testament to the repetitive, adaptive learning scenarios proffered by AI technology. ChatGPT’s proficiency in tailoring educational content to individual performance metrics propels a more incisive and efficacious learning journey. Furthermore, the on-site, real-time feedback from evaluators is likely instrumental in consolidating clinical skillsets, echoing findings on the potency of immediate feedback in clinical education [15, 16].

The study’s strength lies in its pioneering exploration of ChatGPT in pediatric education and the structured use of Mini-CEX for appraising clinical competencies, but it is not without limitations. The ceiling effect may have masked subtle differences in theoretical knowledge, and our small, single-center cohort limits the generalizability of our findings. The transitory nature of the study precludes assessment of long-term retention, a factor that future research should aim to elucidate [17, 18].

Moreover, the ongoing evolution of AI and medical curricula necessitates continuous reevaluation of ChatGPT’s role in education. Future studies should explore multicenter trials, long-term outcomes, and integration strategies within existing curricula to provide deeper insights into AI’s role in medical education. Ethical and practical considerations, including data privacy, resource allocation, and cost, must also be carefully navigated to ensure that AI tools like ChatGPT are implemented responsibly and sustainably.

In conclusion, ChatGPT’s incorporation into pediatric training did not significantly affect the acquisition of theoretical knowledge but did enhance clinical skill development. The high levels of trainee satisfaction suggest that ChatGPT is a valuable adjunct to traditional educational methods, warranting further investigation and thoughtful integration into medical curricula.

## Data Availability

All data sets generated for this study were included in the manuscript.
